# Impact of lifestyle and psychological stress on the development of early onset breast cancer

**DOI:** 10.1097/MD.0000000000005529

**Published:** 2016-12-16

**Authors:** Ping Li, Jialing Huang, Huina Wu, Cuixia Fu, Yun Li, Jiajia Qiu

**Affiliations:** a Department of Nursing Administration, Shanghai Cancer Center; b Department of Oncology, Shanghai Medical College, Fudan University, Shanghai, China.

**Keywords:** early onset breast cancer, lifestyle, psychological stress

## Abstract

The present study aimed to investigate risk factors for early onset breast cancer that are related to lifestyle and psychological stress. A comparative case–control study was performed among patients from the Department of Breast Surgery in Shanghai Cancer Center of Fudan University. The information regarding risk factors associated with the development of early onset breast cancer was collected using a questionnaire in a face-to-face interview. The distribution of the risk factors associated with the development of early onset breast cancer between the patient group and the control group was analyzed with logistic regression. A total of 582 cases of young patients (≤40 years old) with breast cancer and 540 controls of young patients (≤40 years old) with benign breast disease were included in this study. The risk factors for breast cancer in young women include age at first birth (odds ratio [OR] = 0.93, 95% confidence interval [CI]: 0.88–0.98), history of breast cancer in an immediate family member (OR = 2.36, 95% CI: 1.14–4.89), history of genital surgery (OR = 2.11, 95% CI: 1.16–3.82), active and passive smoking in daily life or the environment (OR = 1.64, 95% CI: 1.19–2.25), weekly intake of soy products (OR = 1.24, 95% CI: 1.02–1.49), use of household cooking oil (OR = 2.04, 95% CI: 1.04–4.00), disharmonious marital status (OR = 1.16, 95% CI: 1.06–1.26), frequent depression (OR = 1.32, 95% CI: 1.00–1.75), and negative emotional experiences (OR = 1.15, 95% CI: 1.03–1.29). Our study could provide the basis for risk assessment and preventive interventions for early onset breast cancer.

## Abstract 2

Supplemental Digital Content is available in the text

## 1 Introduction

The worldwide incidence of breast cancer in young patients shows an obvious upward trend in recent years. Based on the data of Surveillance, Epidemiology, and End Results, approximately 11% of breast cancer patients are 35 to 45 years old.^[1]^ The Asian population has a significantly higher proportion of young patients with breast cancer than the Western population, and 9.5% to 12% of all patients with breast cancer in Asia are within this young age range.^[2]^ Recently, the rate of breast cancer diagnosis has rapidly increased in young women in China, and the mortality rate among these young patients is higher than among those who are older. Statistics released by the International Agency for Research on Cancer have shown that patients younger than 40 years accounted for 12.56% of all cancer diagnoses in China in 2008.^[3]^ According to statistics of the Shanghai Center for Disease Control and Prevention, patients <40 years old accounted for 10% to 20% of the total cases of breast cancer in Shanghai from 1990 to 2007.^[4]^ The poor prognosis of early onset breast cancer has gradually attracted widespread international concern in recent years.^[3,5]^

Some countries with a high incidence of breast cancer have made significant progress identifying risk factors for breast cancer that are specific to certain regions and certain populations, which allows doctors to collect relevant information to quantitatively predict the risk of breast cancer for an individual woman.^[6]^ Case–control studies from different regions of China have shown that the risk factors for breast cancer are mainly biological (age at menarche, duration of breast-feeding, menopausal status, and history of contraceptive drug use), psychological (mental stress, depression, negative life events, and long-term depression), and social (passive smoking and residential environmental pollution) factors. Yan et al^[7]^ conducted a meta-analysis of nearly 10 years of Chinese literature to elucidate the relationship between psychological factors and breast cancer and showed that the development of breast cancer is the consequence of the integrated effect of multiple factors including biological, psychological, and social factors. Currently, research on risk factors for early onset breast cancer in China is lacking. Given the gradual increase in the incidence of early onset breast cancer, research investigating the lifestyle and status of young patients prior to disease onset is being performed to identify factors associated with early onset breast cancer. This study aimed to investigate the main physiological and psychological risk factors for breast cancer in young patients based on the biopsychosocial medical model and to explore the impact of lifestyle and psychological stress on the development of early onset breast cancer, thereby providing a basis for the development of relevant prevention strategies.

## 2 Materials and methods

### 2.1 Ethical approval

The present study was approved by the Scientific and Ethical Committee of the Shanghai Cancer Center, Fudan University. Written informed consent was obtained from all participants. The individuals in the present study have given written informed consent to publish these details and data.

### 2.2 Study population and data collection

The present study took place in 1 cancer center in Shanghai between May 2013 and May 2015. Based on a convenient sampling strategy (nonprobability sampling), participants were screened and selected by the principal researcher according to inclusion and exclusion criteria. Patients younger than 40 years of age who had breast disease and who were admitted to the Department of Breast Surgery at Shanghai Cancer Center of Fudan University were included in this study. The patient group consisted of breast cancer patients, while the control group consisted of patients with benign breast disease.

For the patient group, the inclusion criteria were as follows: pathological diagnosis of breast cancer, elementary education or higher, ≤40 years old, no past or current mental illness or unconsciousness, and agreement to participate in the study. The exclusion criteria were as follows: systemic metastasis and presence of arthritis, severe cardiovascular disease, diabetes, or brain dysfunction.

For the control group (the control cases in the hospital), the inclusion criteria were as follows: agreement and ability to participate in the survey, elementary education or higher, ≤40 years old, no past or current mental illness or unconsciousness, and pathological diagnosis of benign breast disease. The exclusion criterion was as follows: presence of arthritis, severe cardiovascular disease, diabetes, or brain dysfunction.

Eligible women were identified and approached by the researchers in our department. Then the researchers would explain details of the study to each patient, and ask for each patient's informed consent to participate in the study. If consent was given, the researchers would distribute the questionnaires to them and notify them that the questionnaires aimed to investigate the status of the patients before they were ill.

### 2.3 Study methods

The present case–control study was designed to collect data regarding risk factors associated with early onset breast cancer and explored the impact of lifestyle and psychological stress on the development of early onset breast cancer.

According to Professor Engel's biopsychosocial model of modern medicine, which attributes the cause of a disease to integrated factors beyond a single biological agent and highlights the impact of psychological and social factors on human health,^[8]^ a self-designed structured questionnaire (Supplemental file) was used to obtain information from cases and controls covering demographic factors, female reproductive factors, lifestyle factors, and psychological factors.

The demographic factors had 7 items and the female reproductive factors contained 9 items. The lifestyle factors contained 22 items covering 3 dimensions wherein smoking and drinking contained 6 items, living and working habits contained 11 items, and dining contained 5 items.

The psychological factors were investigated by the “Psychosocial Stress Survey for Groups (PSSG)” questionnaire and 13 normal psychological items designed through literature and clinical experience. The “ PSSG” is based on Folkman's stress theory^[9]^ and has been applied to the researches of the pathogenesis of tumor, insomnia and such physical and psychological illness. PSSG contained up to 44 items of 3 assessment levels including life events, emotional experience, and responses to living occurrences and moods. Life events contained such items as death of spouse/relatives, marriage problems (divorce/separation), changes in occupations, financial difficulty, and changes in personal health. Emotional experience contained negative emotion and positive emotion. Negative emotion included fear, worries, depression, nervousness, sorrow, helplessness, etc. Positive emotion included delightfulness, happiness, excitement, etc. Reponses to living occurrences and moods contained positive coping and negative coping. Positive coping included forgetting unhappy things as soon as possible, transferring negative factors into positive factors quickly, trying to find help, changing a different environment, being humorous to any problems, etc. Negative coping included being angry at others, losing temper, always crying, always drinking and smoking, etc. PSSG were believed to have good reliability and validity.^[10]^

Seventeen medical information items were collected by researchers including height, weight, age of menarche, menopause, first birth, family history, etc.

### 2.4 Data analysis

The data were input into SPSS 18.0 software. The indices in questionnaire were compared between the 2 groups using *t* tests (nonpaired, 2-tailed with equal variance) and χ^2^ tests. Unconditional logistic regression model was used to analyze the significant risk factors of early onset breast cancer. Correlation power of all risk factors with breast cancer was evaluated by the odds ratio. The odds ratio represented the odds that an outcome would occur given a particular exposure, compared to the odds of the outcome occurring in the absence of that exposure.

## 3 Results

We contacted 891 young women in the patient group. A total of 582 patients consented and completed the questionnaire (65% acceptance). The most common reason for refusal provided was “not interested in it.” The majority (46%) did not provide a reason. Comparisons were made between 540 patients (60% acceptance) and the 362 refusers with regard to available data. The majority of the control group (51%) did not provide a reason.

So from May 2013 to May 2015, a total of 582 patients who were ≤40 years old and who had breast cancer and 540 patients who were ≤40 years old and who had benign breast disease were included in this study.

Comparison of the demographic factors, reproductive factors, and lifestyle and psychological factors between the patient group and the control group is presented in Table 1. The mean age of patients was 34.51 ± 4.61 years, and the mean age of controls was 30.51 ± 6.65 years. The comparison of the 2 groups showed no statistically significant difference in the age at menarche, the number of abortions, the number of live births, regular menstruation, breast symptoms, alcohol drinking, tea drinking, coffee drinking, daily activities, sleeping time, duration of computer use, work intensity, household food expenses and intake of various types of food (meat, seafood, eggs, mushrooms, whole grains, vegetables, fruits, milk, pickled foods, and fried foods), use of health supplements (except fungus spore powder), household drinking water, and interpersonal relationships (all *P* > 0.05).

**Table 1:**
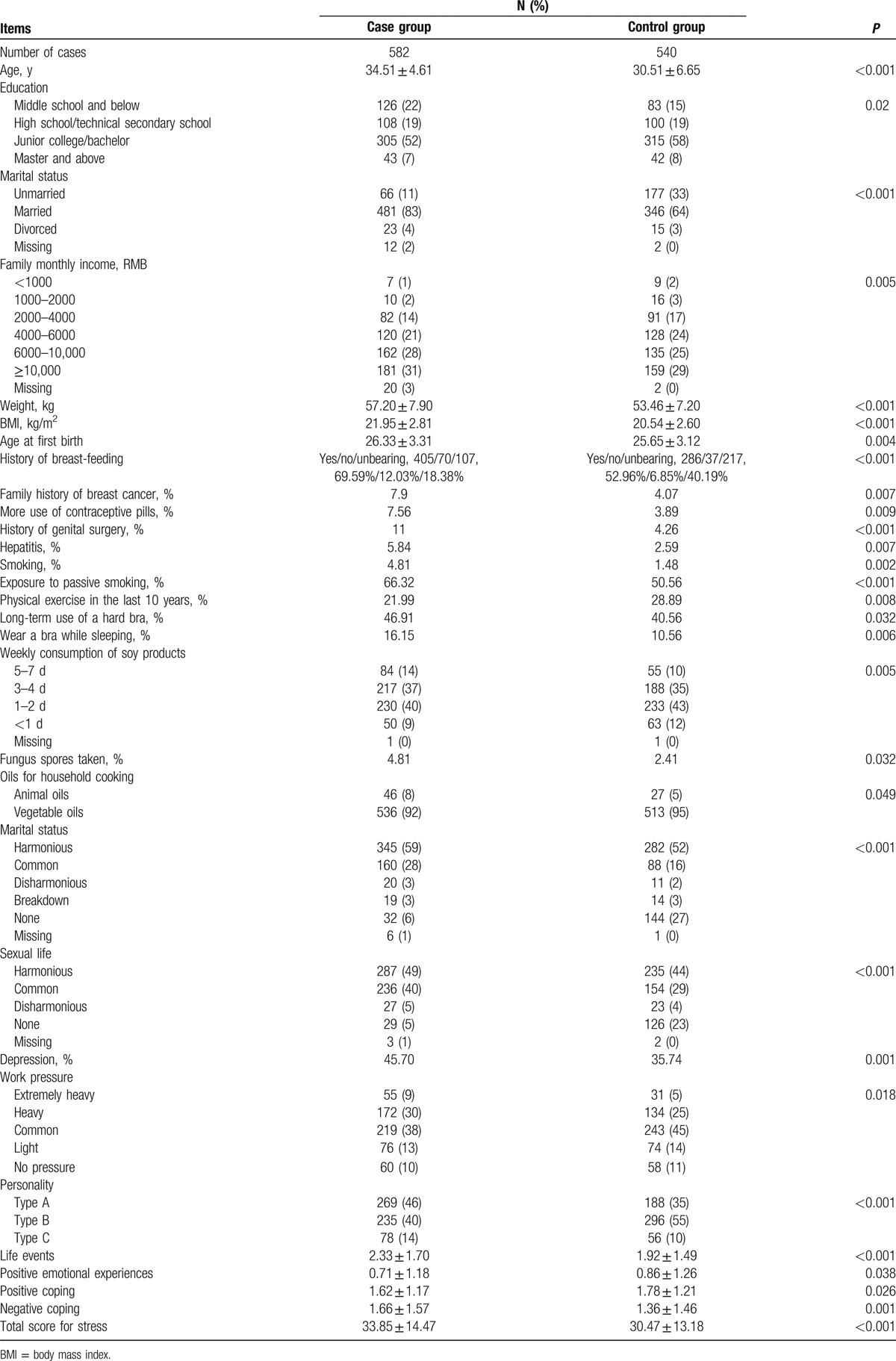
Comparison of the baseline, reproductive factors, and lifestyle and psychological factors between the patient group and the control group.

The results of unconditional logistic regression showed that age at first birth, history of breast cancer in an immediate family member, history of genital surgery, frequent active and passive smoking in daily life or at work, weekly consumption of soy products, use of animal oil for cooking, marital disharmony, frequent depression, and negative emotional experiences were significantly associated with early onset breast cancer (*P* < 0.05) (Table 2). Women who were of a low age at the first birth had 7% lower risk of developing breast cancer than those who were of a higher age at the first birth. Increased risks were attributed to a family history of breast cancer (95% confidence interval [CI]: 1.14–4.89) and a history of genital surgery (95% CI: 1.16–3.82). In addition, exposure to passive smoking in daily life (95% CI: 1.19–2.25), a high weekly consumption of soy products (95% CI: 1.02–1.49), and a high intake of animal oils for household cooking (95% CI: 1.04–4.00) were also associated with an increased risk of breast cancer. With regards to psychological factors, the associations were also found for disharmonious marital status (95% CI: 1.06–1.26), depression (95% CI: 1.00–1.75), and negative emotional experiences (95% CI: 1.03–1.29) with increased breast cancer risk.

**Table 2:**
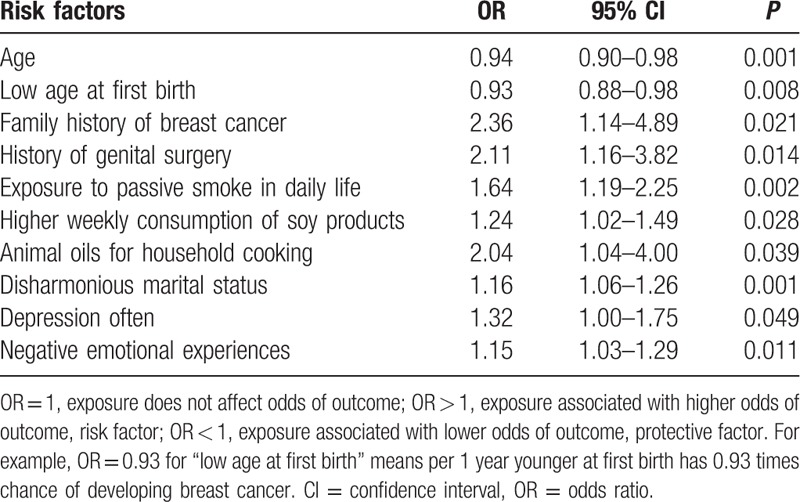
Unconditional logistic regression analysis of the correlation between risk factors.

## 4 Discussion

The present study is a case–control study of the risk factors associated with early onset breast cancer in China.

The logistic regression analysis in the present study showed that the risk of breast cancer in a young woman with a history of breast cancer in an immediate family member was 2.36 times that of a young woman with a negative family history. This finding is consistent with the study of Kilfoy et al.^[11]^ Their cohort study in Shanghai showed that the risk of breast cancer in a woman with a history of breast cancer in an immediate family member was 1.74 times that of a woman with a negative family history, which was more significant for women ≤55 years of age. For young women, they should clearly know about their family history and be aware of their increased risk; then they could pay more attention to their health examination. And health professionals could provide these young women with related specialized information. Epidemiological studies have indicated that the history of reproductive disease increases the risk of breast cancer to varying degrees. The study by Schairer et al^[12]^ showed that those who underwent a hysterectomy alone were at a slightly increased risk of breast cancer. Studies by Cao et al^[13]^ and Ju et al^[14]^ also showed a positive correlation between a history of genital surgery and the incidence of breast cancer. The development of breast diseases is related to estrogen, and estrogen is closely related to breast cancer. Some ovarian diseases can cause abnormal secretion of estrogen.^[13]^ The instability of reproductive factors may be a complex research problem encountered in the study of risk factors for breast cancer. Therefore, regular screening should be provided for young women with a history of genital surgery, and they should be made aware of their increased risk.

The harm to human health caused by smoking is indisputable. Although the rate of active smoking in Chinese women is not high, they are frequently exposed to passive smoking, which is also known as “environmental second-hand smoke.”^[15]^ Studies have confirmed that secondhand smoke is harmful as soon as it is inhaled, and, thus, there is no safe exposure level.^[16]^ Canadian scholars have inferred the causal relationships between passive smoke exposure in nonsmoking young women and the development of breast cancer.^[17]^ Chen et al^[18]^ performed a meta-analysis of passive smoking among Chinese women and found a risk ratio for breast cancer of 1.67 (95% CI: 1.27–2.21) among women exposed to passive smoke. Our study also confirmed that frequent smoke exposure in daily life or at work increased a young woman's risk of breast cancer by 1.64 (95% CI: 1.19–2.25). As a preventable and controllable environmental risk factor, smoking has an important significance for the prevention of early onset breast cancer. Young women should be aware of this environmental factor, try to reduce their exposure to this indirect hazard, and maximize their chances to work and live in good environments.

Studies of soy intake and the risk of breast cancer, including the meta-analyses by Liu et al^[19]^ and Woo et al,^[20]^ have shown that the regular intake of soy products protects against breast cancer. However, our study of young women showed opposite results. Because there are few studies of breast cancer risk among young patients consuming soy products, the results of this single study may not be applicable to all young women in the population. A prospective study would be a better experimental design to explore the correlation between breast cancer and consumption of soy products. Our study also found that using animal fat as a household cooking oil has a risk ratio for breast cancer of 2.04 (95% CI: 1.04–4.00). Animal oil contains more saturated fatty acids and cholesterol. Vegetable oil is the fat obtained from the extraction of the seeds and other parts of plants. Dai et al^[21]^ carried a large population-based case–control study; they found that intake of soybean oil might reduce the risk of breast cancer among women. Vitamin E and phytosterols are the phytochemicals that have been proven to restrain the growth and metastasis of breast cancer in cell culture and animal experiments.^[22]^ Moreover, soybean oil has a high density of essential fatty acids including linoleic and linolenic acid (mostly α-linolenic). The epidemiological evidence from other countries shows that olive oil may reduce the risk of breast cancer.^[23,24]^ Research indicates that the major fatty acid composition of olive oil, which is octadecenoic acid, can block the generation of a protein that may stimulate the growth of breast cancer cells. These findings suggest that cooking oil factor and its interaction with body mechanism may play an important role in the etiology of breast cancer. The results of our study may provide a reference for young women in China to choose a cooking oil. But large-scale experimental researches on cooking oil are still lacking; a prospective experimental study could be better designed to explore the correlation between breast cancer and cooking oil.

Psychological factors, including depression and stress, impair immune function, which, in turn, predisposes an individual to the development of cancer^[25,26]^; it may cause the body to activate human physiological systems in order to maintain stability.^[27]^ Gross et al^[28]^ followed subjects for 24 years to study the relationship between emotions and cancer, and the results showed a statistically significant difference in depression for women with breast cancer, and the level of anxiety also significantly increased the risk of breast cancer. A review by Butow et al^[29]^ found that some case–control studies had shown that emotional depression and alexithymia are strong predictors for the risk of breast cancer in young women. Possel et al^[30]^ also conducted a review focusing on the relationship between depression and the development of breast cancer; 12 of the 15 studies found positive associations between them. Few relevant studies from China are available. A survey by Wang et al^[31]^ of personality traits among breast cancer patients in Southwest China showed an unstable character among patients prior to the diagnosis, which mainly presented as depression, emotion repression, pessimism, and quick anger after stimulation. Our study showed that frequent depression and negative emotional experiences were associated with the development of early onset breast cancer, with risk ratios of 1.32 (95% CI: 1.00–1.75) and 1.15 (95% CI: 1.03–1.29), respectively, which are consistent with the finding that emotional repression increases the risk of breast cancer. The balance of neuroendocrine hormones in women is easily affected by psychological trauma, and long-term adverse emotional experiences may cause hyperplasia in mammary epithelial cells that leads to the development of cancer.^[32]^ Fast-paced urban life and social pressures push young women to pursue quick success without rest, which puts the body into a long-term stressed state that results in exhaustion, physical discomfort, mental depression, lowered self-regulation, and suppression of the body's immunity and endocrine balance. All of these elements may reduce the body's resistance to cancer-causing factors or enhance the body's susceptibility and, thus, may increase the risk of breast cancer. The 10-year follow-up survey of 18,932 women by Nielsen et al^[33]^ found that women working in fast-paced jobs had a higher risk of breast cancer than women working at a proper pace. The correlation of mental state and emotional experiences with breast cancer should draw more attention to young women. Young women should adjust their pace, relax, decompress, and experience positive emotions, which will improve their quality of life and reduce their possibility of illness.

Negative life events and stressful events play an important role in the development of breast cancer.^[34,35]^ A prospective study by Michael et al^[36]^ followed 84,334 women over a period of 7.6 years and found an increased risk of breast cancer among women reporting a negative life event compared to that among women without such an event. Specifically, negative events such as divorce/separation, death of a spouse, and bereavement showed the largest impacts. It is believed that the pathogenesis may be related to hormones or other mechanisms. A case–control study by Eskelinen and Ollonen^[37]^ showed that the score of negative life events and the level of personal pressure among breast cancer patients were significantly different from those who were healthy or had benign disease. Our study showed that the scores of negative life events for the young women in the 2 groups were significantly different, but only marital disharmony was identified in the logistic regression as being a risk factor, with a risk ratio of 1.16 (95% CI: 1.06–1.26). This is consistent with the results of previous studies, which also indicate that harmonious marriage is an important protective factor among young women. A good and stable marriage can promote human health and protect young women by improving their ability to deal with and adapt to life events,^[38]^ and a high-quality marriage requires joint efforts from both partners, so maintaining a harmonious marriage should be a priority.

In summary, in the present case–control study, we found that besides age at first birth, history of breast cancer in an immediate family member, and history of genital surgery, lifestyle and psychological factors play an important role in the risk of breast cancer among young women. The present study was one of the few case–control studies of early onset breast cancer in China. It clarified the relationship of lifestyle factors and psychological factors with early onset breast cancer and indicated the importance of psychological factors in the prevention of early onset breast cancer.

### 4.1 Study limitation

The limitations of the present study include the recruitment and the convenient sample from 1 cancer center. And the numbers between case and control groups do not catch 1:1 match. Maybe they do not represent the majority of young breast cancer patients. Multiple testing can lead to significant findings due to chance.

## Acknowledgments

The authors would like to thank the women who participated in this research and shared their experiences. They would also like to thank Professor Jiayan Huang (Professor of Impact Evaluation & Health Service, School of Public Health, Fudan University) for her suggestions on the present study.

## Supplementary Material

Supplemental Digital Content
