# Supplementary material for: Impact of lifestyle and psychological stress on the development of early onset breast cancer
Source: Medicine (Baltimore). 2016 Dec 16;95(50):e5529. doi: 10.1097/MD.0000000000005529 (PMC5268030; doi:10.1097/MD.0000000000005529)
Supplement: Supplemental Digital Content [file md-95-e5529-s001.doc]

**Risk Factors Questionnaire**

**Part 1 Demographic information**

1. Education: ①middle school and below ②high school/technical secondary school

③junior college and bachelor ④master and above

2. Marital status: ①unmarried ②married ③divorced

3. Number of children:

4. Family monthly income: ①<RMB1000 ②RMB1000～ ③RMB2000～ ④RMB4000～ ⑤RMB 6000～ ⑥≥RMB 10000

5. Current job:

6. The longest career: ①government agency ②medical health ③education system

④public institution ⑤state-owned enterprise ⑥joint ventures or personal ventures

⑦privately operated business ⑧farmer ⑨unemployed ⑩others

7. Any religious belief: ①yes ②no

**Part 2 Female reproductive factors**

1. Have you had mammography before?

①Yes (The latest examination was in / (MM/YY)) ② No

2. What is your frequency for X-ray between 10y-19y?

①Often(every year) ②Seldom(less than 5 times) ③None

3. How do you do contraception?

①Contraceptive pills ②Condoms ③Intrauterine device

4. Do you often use contraceptive pills? ①Yes ②No

5. Have you got breast lumps or thickening?  ①Yes ②No

6. Have you got nipple discharge? ①Yes ②No

7. Have you got nipple itchy or rash? ①Yes ②No

8. Do you have the following gynecological diseases?

①functional uterine bleeding； ②endometrial cancer； ③ovarian cancer

9. Do you have the following conditions?

①history of female genital surgery ②benign diseases of thyroid ③malignant diseases of thyroid ④colorectal cancer ⑤hepatitis

**Part 3 Lifestyle factors**

**(1) Smoking and drinking**

1. Do you smoke? ①No ②used to ③Yes

2. How many cigarettes do you smoke every day? At the age of __I start to smoke.

3. At the age of __I quit smoking.

4. Are you surrounded by the people who always smoke in your daily life or working environment?

①Yes ②No

5. Do you drink? ①No ②less than once a month ③more than once a month

6. What kind of wine do you drink?

①White wine ②Red wine ③Yellow rice wine ④beer ⑤Others

**(2) Living and working habits**

7. Do you drink tea?

①No ②Once a day ③More than twice a day ④Less than 3 times a week

If yes, the tea you drink is ①Black tea ②Green tea ③Oolong ④Others

8. Do you drink coffee?

①No ②once a day ③more than twice a day ④less than 3 times a week

9. Do you have breakfast? ①No ②Occasional ③Often ④Everyday

10. Did you often participate in physical exercises in the last ten years?

①Yes ②No

11. How is your daily activity?

①I am always seated ②Move for 2-4hs a day ③Move for more than 5hs a day

12. How long is your sleep time?

①More than 6hs ②6-8hs ③More than 8hs

13. Time for daily computer use: hours

14. 1) Do you use a hard bra for long? ①Yes ②No

2) You wear bras: ①loose ②tight

3) Do you wear a bra to bed?  ①Yes ②No

15. When did the latest decoration happen for your residence:

16. The intensity of your work (multiple choices): ①Heavy manual labor ②Combined heavy and light labor ③Light manual labor ④Mental work

17. Do you work on night shift? ①Yes ②No

**(3) Diet**

18. The percentage of household food expenses in the whole family income:

①Above 60％ ②50％－59％ ③40％－49％ ④30％－39％ ⑤Below 30％

19. Weekly consumption of following food:

1) Fresh meat: ①5-7days ②3－4days ③1-2days ④Less than one day

2) Fried food: ①5-7days ②3－4days ③1-2days ④Less than one day

3) Pickled food: ①5-7days ②3－4days ③1-2days ④Less than one day

4) Fish, shrimp, seafood: ①5-7days ②3－4days ③1-2days ④Less than one day

5) Eggs: ①5-7days ②3－4days ③1-2days ④Less than one day

6) Fresh soy products: ①5-7days ②3－4days ③1-2days ④Less than one day

7) Fungus & Mushrooms: ①5-7days ②3－4days ③1-2days ④Less than one day

8) Grains, coarse grains: ①5-7days ②3－4days ③1-2days ④Less than one day

9) Vegetables: ①5-7days ②3－4days ③1-2days ④Less than one day

10) Fruits: ①5-7days ②3－4days ③1-2days ④Less than one day

11) Desserts: ①5-7days ②3－4days ③1-2days ④Less than one day

12) Dairy product: ①less than 250ml ②250ml— ③500ml－ ④1000ml－

20.Do you have following health supplements (multiple choices)?

①royal jelly ②Honey ③Placenta ④Hasma ⑤Vit E ⑥Cubilose

⑦Cordyceps sinensis ⑧Ganoderma lucidum spores ⑨Others

21. Household drinking water? ①Tap water ②Non-tap water

22. Household cooking oil: ①Animal oil ②Vegetable oil

**Part 4 Psychological factors**

1. Your marital status: ①Harmonious ②Common ③Disharmonious ④Breakdown

2. Do you live without your closest people for long? ①Yes ②No

3. Your Interpersonal relationships: ①Good ②Normal ③Poor

4. Your sexual life: ①Harmonious ②Common ③Disharmonious

5. Are you always in low spirits? ①Yes ②No

6. Do you always feel depression? ①Yes ②No

7. Your work pressure: ①Extremely heavy ②heavy ③Common ④Light ⑤No pressure

8. Are you easy to be impatient? ①Yes ②No

9. Do you always worry about family problems? ①Yes ②No

10. Are you satisfied with your family status? ①Yes ②No

11. Do you like quiet surroundings? ①Yes ②No

12. Do you like bustling surroundings? ①Yes ②No

13. Your personality:

Type A：Quick temper and impatient. Aggressive and competitive, ambitious with strong entrepreneur spirit. Quick in action, punctuality. Easy to have Mood fluctuations and suspicious. Lack of exercises.

Type B：Easy-going and uncompetitive. Live in a free and unfettered style and without measure for gains and lose. Easy to be satisfied. Take time working and living. The sense of time is not strong.

Type C：Depressive personality of depressed emotions. Afraid of competitions, too submissive, swallow the insult and keep self in a sulk.

1. What significant life events have happened in the last 5 years before your disease?
2. Overwork
3. Death of a loved one
4. Disharmonious status in family or marriage
5. Disharmonious in interpersonal relationships
6. Unfavorable changes in career
7. Financial difficulties
8. Frustrations in the career and study
9. Legal troubles
10. Problems related to children’s future
11. Bad living environment
12. Personal remarkable achievements or happy things
13. Serious illness of family members
14. Changes of personal health
15. What obvious and lasting emotions have you experienced due to the above life events?

Negative Emotion：①Fearful ②Distressed ③Depressed ④Nervous ⑤Angry

⑥Despairing ⑦Anxious ⑧Worried ⑨Sad ⑩Helpless

Positive Emotion：①Glad ②Delighted ③Joyful ④Excited ⑤Relieved

1. How do you cope with such unhappy events and emotions?

Positive coping: ①Forget it as soon as possible; ②Pretend that nothing happened；

③Think positively and let go； ④Compare with people with similar situations；

⑤Turn negative factors into positive factors quickly； ⑥Take the initiative to turn to someone for help or consolation if necessary； ⑦Change an environment to avoid unpleasant emotions； ⑧Tackle problems with humorous attitudes；

Negative coping: ①Easy to grieve over the past and delusion； ②Lose temper and be angry at others； ③Become depressed when troubles come； ④Constrain and hide moods in the heart and cannot get rid of it; ⑤Sometimes want to cry silently for the annoyance； ⑥Smoke or drink alone to drown sorrows； ⑦Toss and turn when something goes wrong； ⑧Like to be alone when feeling distressed；

**Part 4 Medical information (by investigator)**

1. Height: cm Weight: kg

2. Time of surgery:

3. What kind of surgery?

4. Pathology:

5. Any lymphatic node metastases? ①Yes ②No

6. Any distant metastases? ①Yes ②No

7. Fasting plasma glucose:

8. Do you have diabetes? ①Yes ②No

9. Age of menarche:

10. Age of menopause:

11. Age of first birth:

12. Number of abortions: ; Number of live births: ; Number of full production times: ; Number of preterm labor: ;

13. Do you breastfeed? ①Yes ②No ③childless

14. Is your menstruation regular? ①Yes ②No

Duration of your menstruation: ;

15. Have you had breast surgery before? ①Yes ②No

16. Have you had unilateral breast cancer before? ①Yes ②No

17. Has anyone had breast cancer in your direct relatives? ①Yes ②No
